# Research on Trajectory Tracking Control Method for Crawler Robot Based on Improved PSO Sliding Mode Disturbance Rejection Control

**DOI:** 10.3390/s25072113

**Published:** 2025-03-27

**Authors:** Zhiyong Yang, Qing Lang, Yuhong Xiong, Shengze Yang, Changjin Zhang, Lielei Deng, Daode Zhang

**Affiliations:** 1 Engineering Research and Design Institute of Agricultural Equipment, Hubei University of Technology, Wuhan 430068, China; yzy017@126.com; 2 Hubei Engineering Research Center for Intellectualization of Agricultural Equipment, Wuhan 430068, China; 3 School of Mechanical Engineering, Hubei University of Technology, Wuhan 430068, China; 102210057@hbut.edu.cn (Q.L.); xyh010518@126.com (Y.X.); 102312493@hbut.edu.cn (S.Y.); 102310154@hbut.edu.cn (C.Z.); 102312494@hbut.edu.cn (L.D.)

**Keywords:** crawler robot, sliding mode active disturbance rejection control, trajectory tracking, path vector field guidance

## Abstract

To address the issues of low trajectory tracking accuracy and difficulties in tuning control parameters for crawler robots operating in uneven terrains, this paper proposes a trajectory tracking control method. The method is based on improved particle swarm optimization and sliding mode active disturbance rejection control (SPSO-SMADRC). Firstly, considering the influence of disturbances such as terrain undulations and soil inhomogeneity on trajectory deviation, the kinematic and dynamic models of the crawler robot are established. A vector field guidance approach is employed to transform the trajectory tracking task into a heading control problem. The heading angle is adaptively adjusted based on the position deviation and path curvature. A nonlinear extended state observer is introduced to estimate external disturbances. A velocity-based SMADRC controller is designed to dynamically regulate the robot’s linear and angular velocities. This allows real-time correction of the robot’s motion. To overcome the tendency of the standard particle swarm optimization (PSO) algorithm to fall into local optima during controller parameter tuning, a nonlinear dynamic adjustment strategy was adopted. This strategy adaptively adjusts the inertia weight and learning factors, enhancing the algorithm’s global search capability. Comparative experiments were conducted using two types of curved trajectories: U-shaped and V-shaped paths. The experimental results show that, under the proposed SPSO-SMADRC method, the crawler robot achieved maximum position errors of 8.28 cm and 9.26 cm, average position errors of 1.41 cm and 2.94 cm, and maximum heading angle deviations of 0.56 rad and 0.87 rad. The standard deviations of the position errors were 3.19 and 4.28, respectively. Compared with conventional PSO-based SMADRC and standard SMADRC methods, the proposed approach improved the navigation tracking accuracy. In the U-shaped trajectory, the maximum position error was reduced by 19.22% and 38.21%, the average position error by 40.00% and 65.53%, and the heading angle error by 28.21% and 74.66%. In the V-shaped trajectory, the maximum position error was reduced by 17.39% and 38.95%, the average position error by 51.71% and 52.04%, and the heading angle error by 80.58% and 84.49%. These results demonstrate that the proposed SPSO-SMADRC method significantly enhances trajectory tracking performance and system robustness. It provides effective support for high-precision autonomous navigation of crawler robots in complex and unstructured environments.

## 1. Introduction

In typical tasks such as agricultural inspection, disaster rescue, field inspection, etc., crawler robots are widely used for autonomous operations in complex environments by virtue of their good obstacle-crossing performance and terrain adaptation capability [1]. With the development of automatic navigation technology, the continuous improvement of key technologies such as localization and posture measurement, path planning, and motion control has significantly improved the operational accuracy and stability of tracked robots in complex terrain [2,3]. However, in non-flat terrain, due to the problems of large ground undulation, uneven attachment conditions, and frequent external disturbances, tracked robots are still prone to operational problems such as trajectory deviation, lagging system adjustment response, and even attitude destabilization, which seriously affects their operational efficiency and mission reliability. Therefore, improving the trajectory tracking accuracy and adaptability of crawler robots to complex terrain has become one of the key technologies to ensure their efficient and stable operation in practical applications.

Currently, with the continuous expansion of robot application scenarios, researchers have proposed various trajectory tracking control methods to address challenges in different environments, including PID control [4,5], pure tracking control [6,7], active disturbance rejection control (ADRC) [8,9], sliding mode control (SMC) [10,11], and model predictive control (MPC) [12,13]. Mai et al. [14] designed an adaptive fuzzy PID controller for tracking robot speed control and steering, utilizing backstepping control to eliminate posture deviations, significantly improving trajectory tracking performance. Ji et al. [15] designed an adaptive second-order sliding mode (ASOSM) control system and incorporated a preview lateral offset model to enhance the trajectory tracking accuracy of unmanned tractors in unstructured environments. However, existing control methods still have certain limitations in terms of tracking real-time performance and adaptability to complex environments. Hou et al. [16] successfully combined their method with constrained model predictive control (MPC) and global terminal sliding mode control (GTSMC), improving the trajectory tracking performance of underactuated systems. To better assess the centroid offset angle and yaw rate, Li et al. [17] achieved high-precision trajectory tracking of robots by integrating the cubic Kalman algorithm with fuzzy compensation and preview angle control. Li et al. [18] introduced an offset rate analysis and compensation mechanism under the MPC framework to improve the trajectory tracking accuracy of underwater crawler bulldozers. However, when crawler robots operate on land, they often require more position constraints due to complex terrains. To better address path deviations caused by complex terrain during trajectory tracking, Chen et al. [19] studied the relationship between global path parameters and sliding mode control to enable the robot to converge stably along the target path by introducing a prescribed performance vector field in combination with sliding mode control. Kong et al. [20] designed a flexible and adjustable parameter function by combining a self-resilient controller with an improved guidance vector field (GVF) to enhance the robot path tracking performance and obstacle avoidance capability. Although these methods have made significant progress in the field of trajectory tracking, the adaptability in different operational scenarios still needs to be improved because the control parameters need to be manually adjusted.

To address the difficulties of manual tuning of controller parameters and poor adaptability, intelligent optimization parameter algorithms have been widely applied in the field of trajectory tracking control. Li et al. [21] used particle swarm optimization (PSO) to optimize the trajectory tracking controller parameters for four-wheel steering vehicles (4WS). Gökçe et al. [22] applied PSO to optimize the PID controller parameters, solving the path tracking and stability control issues of four-wheel steering agricultural robots during slippage. However, in previous studies, the inertia weight and learning factors of the PSO algorithm were typically constants, which caused the algorithm to fall into local optima. To address this, Qiao et al. [23] employed a particle swarm algorithm with an adaptive change in inertia weight to optimize PID controller parameters, significantly reducing path tracking deviation compared to manual tuning. Guo et al. [24] used particle swarm optimization (PSO) to optimize a safe speed adjustment mechanism in global path planning, which solves the local minimum problem well and significantly improves the global optimization performance of path planning. Feng et al. [25] proposed an improved particle swarm optimization (PSO) algorithm to optimize the PID controller parameters through nonlinear adaptive inertia weights, asynchronous learning coefficient strategy, and elite mutation method to improve the trajectory tracking accuracy.

The above methods provide an effective solution to the trajectory tracking control problem by predicting and compensating for possible robot offsets as well as external perturbations. However, the lack of constraints on the position of the robot in the case of tracked robots slipping due to drastic terrain changes affects the accuracy of trajectory tracking. In addition, the system’s ability to regulate in response to strong terrain changes is still deficient, resulting in limited control accuracy and motion stability of the robot in complex environments.

Aiming at the above problems, this paper designs a sliding mode self-resistant trajectory tracking control method based on the optimized parameters of the improved particle swarm algorithm, which solves the problems of the crawler robot’s difficulty in parameter adjustment and insufficient compensation for external perturbations in complex terrain, and provides a reliable solution for the high-precision trajectory tracking of crawler robots in complex environments. The specific methods are as follows:

(1) Establish the motion model of crawler robot through theoretical analysis, fully consider the influence of terrain and system dynamics characteristics, and lay the theoretical foundation for trajectory tracking.

(2) Based on mathematical modeling, a sliding mode self-imposed anti-disturbance control (SMADRC) strategy is designed, which introduces position constraints in combination with the path vector field and compensates for external perturbations in real time, dynamically correcting the robot’s speed to ensure stable tracking in complex terrain.

(3) An improved particle swarm optimization (PSO) algorithm is introduced to optimize the controller parameters to enhance the self-adaptive ability of the system, avoiding the tediousness of manual parameter tuning and improving the robustness of trajectory tracking and environmental adaptability.

(4) The effectiveness and superiority of the proposed method are verified by tracking experiments under different trajectories.

## 2. Crawler Robot Model

### 2.1. Crawler Robot Kinematic Model

**Figure 1:**
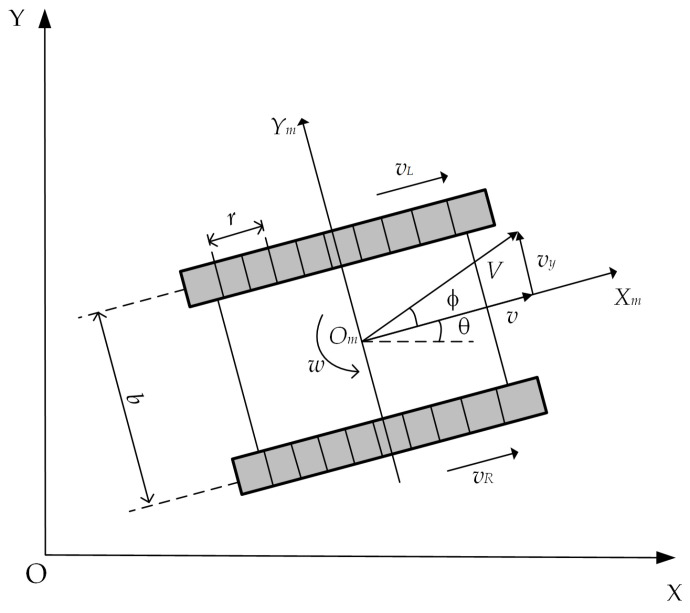
Crawler robot kinematic model.

This study focuses on a crawler robot with dual independent motor drives. Based on the differential steering characteristics, the kinematic model shown in Figure 1 is established. The following assumptions are made to simplify the motion characteristics of the crawler robot: (1) The robot’s center of mass coincides with the geometric center at a single point; (2) The elastic deformation and minor deformation between the tracks and the ground are neglected. The parameter *b* represents the wheelbase of the crawler robot, and *r* is the radius of the drive wheels. The robot’s position in the global coordinate system *XOY* is described by the coordinates (*x*, *y*, *θ*), where *x* and *y* are the horizontal and vertical coordinates of the robot’s center of mass in the global coordinate system, and *θ* represents the robot’s body longitudinal angle relative to the *X*-axis, indicating the heading angle. The body coordinate system *X_m_O_m_Y_m_* is established at the geometric center *O_m_* of the crawler robot. The forward speed of the robot along the body’s forward axis is *ν*, and *ν_y_* represents the lateral velocity the robot may experience due to external disturbances. *ω* is the angular velocity of the body. Additionally, considering potential deviations caused by complex terrain, the deviation angle *ϕ* is defined to represent the offset between the robot’s actual velocity and the forward direction.

Thus, the kinematic model of the crawler robot in the global coordinate system is established as follows:

(1) $$\left[\begin{array}{c} \dot{x}\\ \dot{y}\\ \dot{\theta } \end{array}\right]=\left[\begin{array}{ccc} \cos\theta & -\sin\theta & 0\\ \sin\theta & \cos\theta & 0\\ 0 & 0 & 1 \end{array}\right]\left[\begin{array}{c} v\\ v_{y}\\ \omega \end{array}\right]=\left[\begin{array}{ccc} \cos\phi \cos\theta & -\sin\phi \sin\theta & 0\\ \cos\phi \sin\theta & \sin\phi \cos\theta & 0\\ 0 & 0 & 1 \end{array}\right]\left[\begin{array}{c} V\\ \omega \end{array}\right]$$

When the crawler robot steers, the theoretical velocities of the left and right tracks, *v_L_* and *v_R_*, are shown in Equation (2):

(2) $$\left\{\begin{array}{l} v_{L}\\ v_{R} \end{array}\right.=\left\{\begin{array}{l} \eta \cdot \frac{60\cdot l\cdot a}{2\pi \cdot N_{L}}\\ \eta \cdot \frac{60\cdot l\cdot a}{2\pi \cdot N_{R}} \end{array}\right.$$

The actual velocities *v_l_* and *v_r_* are shown in Equation (3):

(3) $$\left\{\begin{array}{l} v_{l}\\ v_{r} \end{array}\right.=\left\{\begin{array}{l} v-\frac{\omega \cdot b}{2}\\ v+\frac{\omega \cdot b}{2} \end{array}\right.$$

The offset angle *ϕ* can be expressed as in Equation (4), in order to describe the potential trajectory deviations that may occur during the robot’s actual motion, thereby improving the accuracy of the kinematic model and providing a reliable reference for the control algorithm.

(4) $$\phi =\arctan\frac{v_{r}-v_{l}}{b}-\arctan\frac{v_{R}-v_{L}}{b}$$

### 2.2. Crawler Robot Dynamics Model

When operating in uneven environments, the crawler robot is influenced by various unknown factors, making its motion characteristics difficult to predict with high accuracy. This is particularly true during transient steering, where the left and right tracks may experience uneven forces due to external disturbances such as slopes, obstacles, or uneven terrain, further impacting the robot’s motion stability and trajectory tracking precision.

**Figure 2:**
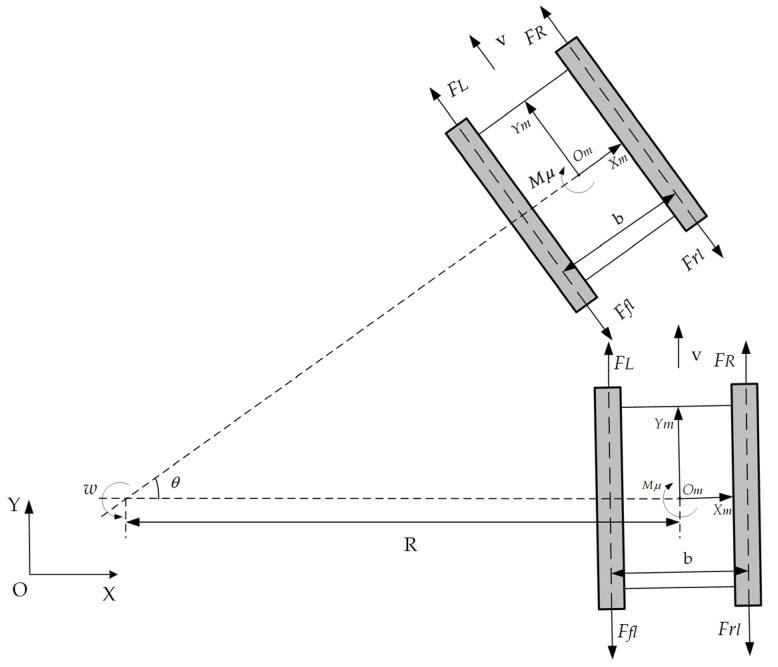
Steering force analysis of the crawler robot.

Based on the established plane coordinate system and the robot’s coordinate system, a force analysis of the moving crawler robot is conducted, as shown in the Figure 2. *O_m_* represents the robot’s steering center, and *R* is the robot’s steering radius. *F_R_* and *F_L_* are the effective traction forces acting on the robot, *F_f_* is the frictional force, *F_c_* is the uncertain longitudinal resistance force, *J* is the robot’s moment of inertia, *M_μ_* is the steering resistance torque, and *M_c_* is the uncertain resistance torque.

By analyzing the force situation of the crawler robot in Figure 2, the robot’s dynamic equation is obtained based on the force and torque balance equations:

(5) $$\left\{\begin{array}{l} m{\dot{\nu }}_{c}=F_{L}+F_{R}-F_{fl}F_{fr}-F_{c}\\ J{\dot{\omega }}_{C}=(F_{R}-F_{L})\cdot d-M_{\mu }-M_{c} \end{array}\right.$$

where *d* = *b*/2, and the frictional resistance and steering resistance torque acting on the robot are shown in Equation (6):

(6) $$\left\{\begin{array}{l} F_{fl}=F_{fr}=\frac{fmg}{2}\\ M_{\mu }=\frac{\mu mgL}{4} \end{array}\right.$$

where *L* is the track–ground contact length, f is the ground friction coefficient, and μ is the maximum ground steering coefficient, which is related to the ground conditions.

The dynamic relationship between the effective traction force on the crawler robot’s drive wheels and the motor can be expressed by the following Equation:

(7) $$\left\{\begin{array}{l} J_{e}{\dot{\omega }}_{l}+B_{e}\omega_{l}=t_{l}-F_{L}r\\ J_{e}{\dot{\omega }}_{r}+B_{e}\omega_{r}=t_{r}-F_{R}r \end{array}\right.$$

Considering the velocity relationships $v=\frac{(\omega_{r}+\omega_{l})r}{2}$ and $\omega =\frac{(\omega_{r}-\omega_{l})r}{2d}$, based on Equations (5)–(7), the dynamic model of the mobile robot with the motor torque as the control variable is obtained as:

(8) $$\left\{\begin{array}{l} \dot{\nu }=-\frac{2B_{e}}{mr^{2}+2J_{e}}\nu +\frac{r}{mr^{2}+2J_{e}}(t_{r}+t_{l})-2\mu g-\frac{F_{c}}{m}\\ \dot{\omega }=-\frac{2B_{e}}{Jr^{2}+2J_{e}d^{2}}\omega +\frac{rb}{mr^{2}+2J_{e}d^{2}}(t_{r}-t_{l})-\frac{\mu mgL}{2J}-\frac{M_{c}}{2J} \end{array}\right.$$

Furthermore, if the coil inductance voltage of the motor is neglected, the simplified DC servo motor model can be expressed as:

(9) $$\left\{\begin{array}{l} \tau_{l}=\frac{k_{a}(u_{l}-k_{b}\omega_{l})}{R_{a}}\\ \tau_{r}=\frac{k_{a}(u_{r}-k_{b}\omega_{r})}{R_{a}} \end{array}\right.$$

where *u_l_* and *u_r_* are the voltages applied to the left and right wheel motors; *k_a_* and *k_b_* are the motor’s torque constant and voltage constant, respectively; *R_a_* represents the resistance of the motor coil; and the above parameters have taken into account the effect of the transmission ratio.

Consider the following relationship:

(10) $$\left\{\begin{array}{l} u_{v}=\frac{u_{r}+u_{l}}{2}\\ u_{\omega }=\frac{u_{r}-u_{l}}{2} \end{array}\right.$$

Substituting Equations (9) and (10) into Equation (8), the disturbances that the crawler robot may encounter during actual motion, represented as $\zeta_{\nu }$ and $\zeta_{\omega }$, can be expressed. The robot’s dynamic model can then be represented as:

(11) $$\left[\begin{array}{c} \dot{\nu }\\ \dot{\omega } \end{array}\right]=\left[\begin{array}{cc} a_{1} & 0\\ 0 & a_{2} \end{array}\right]\left[\begin{array}{c} \nu \\ \omega \end{array}\right]+\left[\begin{array}{cc} b_{1} & 0\\ 0 & b_{2} \end{array}\right]\left[\begin{array}{c} u_{v}\\ u_{\omega } \end{array}\right]-\left[\begin{array}{c} -2\mu g\\ \frac{\mu mgL}{2J} \end{array}\right]+\left[\begin{array}{c} \zeta_{\nu }\\ \zeta_{\omega } \end{array}\right]$$

In Equation (11):

$$a_{1}=-\frac{2B_{e}R_{a}+2k_{a}k_{b}}{\left(mr^{2}+2J_{e}\right)R_{a}},a_{2}=-\frac{2B_{e}R_{a}+2k_{a}k_{b}d^{2}}{\left(Jr^{2}+2J_{e}d^{2}\right)R_{a}},b_{1}=\frac{2rk_{a}}{\left(mr^{2}+2J_{e}\right)R_{a}},b_{2}=\frac{2rdk_{a}}{\left(mr^{2}+2J_{e}d^{2}\right)R_{a}}$$

## 3. Trajectory Tracking Control Scheme

**Figure 3:**
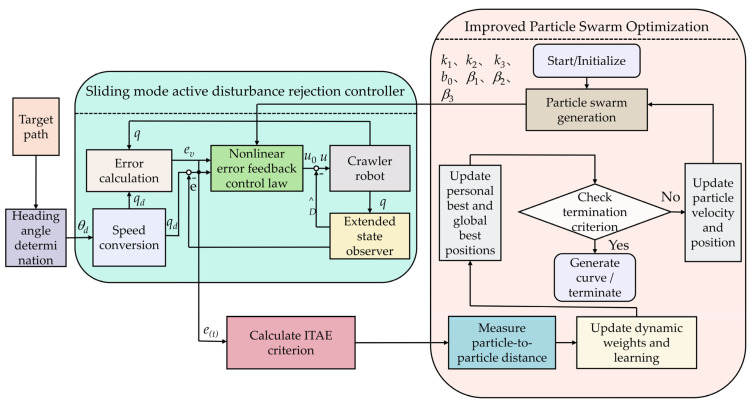
Crawler robot trajectory tracking control scheme.

Due to the complexity and variability of uneven terrain, crawler robots often experience trajectory tracking errors, reducing tracking accuracy. To address this issue, this paper combines path vector field guidance and sliding mode active disturbance rejection control (SM-ADRC) to design an adaptive disturbance rejection trajectory tracking controller, which minimizes tracking errors. The overall control system framework is illustrated in Figure 3. Utilizing path vector field guidance, the desired heading angle is generated from the input trajectory. Based on the crawler robot’s real-time position and desired position, a velocity control command is generated and used as an input to guide motion. Under the SM-ADRC framework, a nonlinear velocity tracking controller is designed using velocity error and velocity dynamic estimation error, producing the initial control input. Subsequently, an extended state observer (ESO) estimates disturbances in real time and provides compensation, ultimately yielding the disturbance-compensated control input, ensuring stable trajectory tracking. Furthermore, a self-adaptive particle swarm optimization (SPSO) algorithm dynamically tunes key control parameters, improving disturbance rejection and control performance.

### 3.1. Heading Angle Determination Based on Path Vector Field

When operating in uneven environments, the crawler robot often experiences deviations between the actual and target trajectories due to the lack of positional constraints, especially when encountering complex terrain or external disturbances. To address this issue, this paper establishes a path vector field around the desired path and introduces directional information, enabling the robot to more effectively return to the path when deviating.

**Figure 4:**
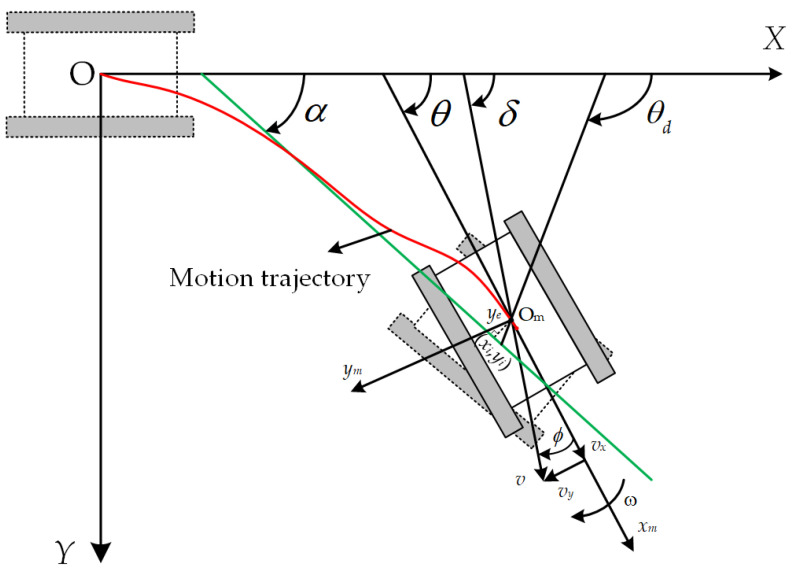
Crawler robot operating trajectory diagram. The red line is the running trajectory and the green line is the tangent to the target trajectory.

To analyze the trajectory movement of the crawler robot in uneven environments, as shown in Figure 4, an inertial coordinate system *XOY* and a body coordinate system *X_m_O_m_Y_m_* are established. The *X_m_* axis points in the direction of the robot’s forward movement, with the robot’s forward linear velocity denoted as *ν*. The variables *ν_x_*, *ν_y_*, and *ω* represent the robot’s longitudinal speed, lateral speed, and angular velocity in the body coordinate system, respectively. Assume the current position of the crawler robot is (*x_i_*,*y_i_*), *θ* is the robot’s heading angle, and *P*(*x_p_,y_p_*) is the nearest point on the trajectory to the current position. *y_e_* is the lateral error between the robot and the target path. *α* is the angle between the tangent of the target trajectory and the positive direction of the *X*-axis in the inertial coordinate system. *δ* is the robot’s heading angle, *ϕ* is the deviation angle that the robot may experience in complex environments, *δ* = *θ* + *ϕ*, and *θ_d_* is the desired heading angle, determined using the vector field guidance method.

The pose and velocity of the target trajectory point are represented as the vector *Pr* = [*x_r_*,*y_r_*,*θ_r_*], and the trajectory tracking error equation of the crawler robot in the local coordinate system is established as:

(12) $$\left[\begin{array}{c} x_{e}\\ y_{e}\\ \theta_{e} \end{array}\right]=\left[\begin{array}{ccc} \cos & \sin\theta & 0\\ -\sin\theta & \cos\theta & 0\\ 0 & 0 & 1 \end{array}\right]\left[\begin{array}{l} x_{r}-x_{i}\\ y_{r}-y_{i}\\ \theta_{r}-\theta_{i} \end{array}\right]$$

Taking the derivative of Equation (12) and substituting Equation (1) gives:

(13) $$\left[\begin{array}{c} {\dot{x}}_{e}\\ {\dot{y}}_{e}\\ {\dot{\theta }}_{e} \end{array}\right]=\left[\begin{array}{l} V\sin(\theta -\alpha +\phi )\\ V\cos(\theta -\alpha +\phi )\\ \;\omega_{r}-\omega \; \end{array}\right]$$

**Figure 5:**
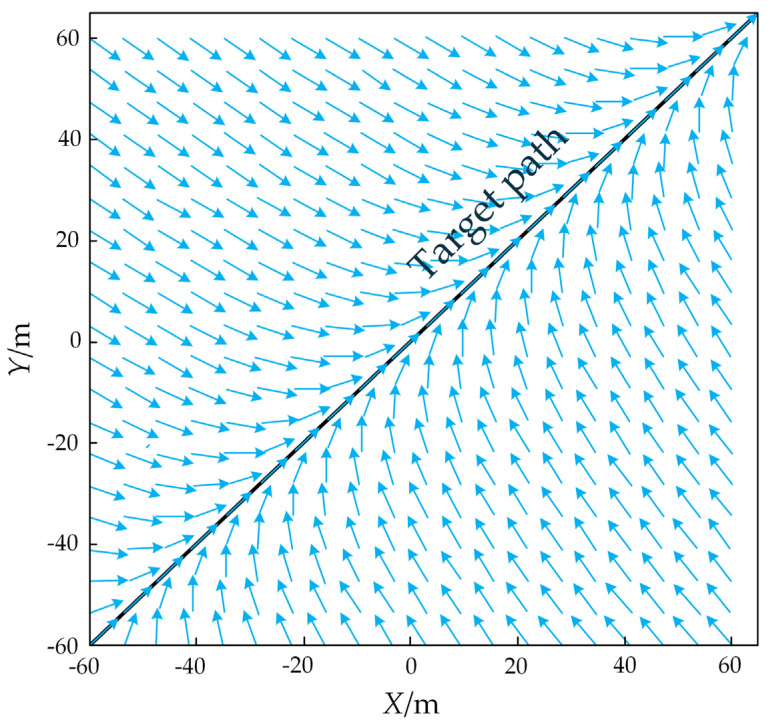
Path vector field. The arrow represents the ensemble vector pointing to the path.

To achieve trajectory tracking for the crawler robot, a vector field is constructed to guide the robot toward the target path. A guiding vector pointing to the desired motion direction is generated at the robot’s current position. This method combines the tangent vector field of the target path direction with the normal vector field perpendicular to the target path, forming a smoothly transitioning global vector field. This guides the crawler robot to automatically adjust its running direction in complex terrain. The schematic diagram of the path vector field is shown in Figure 5.

First, find the closest point on the target path to the current position of the robot *P*(*x_r_*,*y_r_*):

(14) $$p=\arg\min\sqrt{{\left(x(i)-x(r)\right)}^{2}+{\left(y(i)-y(r)\right)}^{2}}$$

The Euclidean norm of the trajectory vector is set as follows:

(15) $$\left\{\begin{array}{l} ∥D1∥=\frac{s}{∥s∥}\\ ∥D2∥=∥\lambda_{1}\cdot \frac{\lambda_{2}|y_{e}|}{1+\lambda_{2}|y_{e}|}∥ \end{array}\right.$$

where

(16) $$\left\{\begin{array}{l} s=\left[\begin{array}{l} x_{i}-x_{r}\\ y_{i}-y_{r} \end{array}\right]\\ ∥s∥=\sqrt{{\left(x_{r}-x_{i}\right)}^{2}-{\left(y_{r}-y_{i}\right)}^{2}} \end{array}\right.$$

The vector field formula can be expressed as:

(17) $$D=D_{1}+D_{2}$$

After superimposing, the direction vector generated by the vector field at the crawler robot’s current position represents the expected velocity direction of the robot at that position.

The heading angle can be expressed as:

(18) $$\delta_{d}=\alpha -\mathrm{sign}(y_{e})\arctan(\lambda_{1}\cdot \frac{\lambda_{2}|y_{e}|}{1+\lambda_{2}|y_{e}|})$$

where *λ*_1_ = 20 and *λ*_2_ = 0.3.

The desired forward direction angle is:

(19) $$\theta_{d}=\delta_{d}-\phi$$

Assuming that the forward direction angle of the crawler robot can track the desired direction angle, *θ* = *θ_d_*, by substituting Equations (18) and (19) into Equation (12), the position error is obtained as follows:

(20) $$\begin{array}{l} {\dot{y}}_{e}=U\sin(\theta -\alpha +\phi )\\ \;=U\sin(\theta_{d}-\alpha +\phi )\\ \;=U\sin(\delta_{d}-\alpha )\\ \;=U\sin[-sign(y_{e})\arctan(\lambda_{1}\cdot \frac{\lambda_{2}|y_{e}|}{1+\lambda_{2}|y_{e}|})]\\ \;=-U\cdot sign(y_{e})\cdot \sin[\arctan(\lambda_{1}\cdot \frac{\lambda_{2}|y_{e}|}{1+\lambda_{2}|y_{e}|})]\\ \;=-U\cdot sign(y_{e})\cdot \frac{\lambda_{1}\cdot \frac{\lambda_{2}|y_{e}|}{1+\lambda_{2}|y_{e}|}}{\sqrt{1+(\lambda_{1}\cdot \frac{\lambda_{2}|y_{e}|}{1+\lambda_{2}|y_{e}|}{)}^{2}}} \end{array}$$

To prove the stability of the trajectory guidance law, the following Lyapunov function is chosen:

(21) $$V_{1}=\frac{1}{2}y_{e}^{2}$$

Taking the derivative of Equation (21) and substituting Equation (20) yields:

(22) $$\begin{array}{l} {\dot{V}}_{1}=y_{e}{\dot{y}}_{e}\\ \;=-U\cdot y_{e}\cdot sign(y_{e})\cdot \frac{\lambda_{1}\cdot \frac{\lambda_{2}|y_{e}|}{1+\lambda_{2}|y_{e}|}}{\sqrt{1+(\lambda_{1}\cdot \frac{\lambda_{2}|y_{e}|}{1+\lambda_{2}|y_{e}|}{)}^{2}}}\\ \;=-U\cdot \frac{\lambda_{1}\cdot \frac{\lambda_{2}{y_{e}}^{2}}{1+\lambda_{2}|y_{e}|}}{\sqrt{1+(\lambda_{1}\cdot \frac{\lambda_{2}|y_{e}|}{1+\lambda_{2}|y_{e}|}{)}^{2}}}\leq 0 \end{array}$$

According to Lyapunov stability theory, the position error *y_e_* gradually converges to zero over time, indicating that the system is asymptotically stable.

### 3.2. Speed Sliding Mode Self-Disturbance Control

To address the issue of trajectory tracking control degradation due to model inaccuracies caused by external disturbances when the crawler robot operates in uneven terrain, a sliding mode disturbance rejection velocity tracking controller is designed. The controller outputs control quantities *u*_0_ = (*u*_1_,*u*_2_) based on the deviation between the desired speed *q_d_* = (*ν_d_*,*ω_d_*) and the real-time feedback speed *q* = (*ν*,*ω*). The controller introduces a nonlinear extended state observer (ESO) to estimate the total disturbance in the system and incorporates this disturbance as a compensation term. This term is combined with the control signal to yield the actual control quantity *u* = (*u_v_*,*u_ω_*), which dynamically adjusts the left and right track speeds to maintain directional stability.

To ensure that the robot pose error system in Equation (12) asymptotically converges to zero, the velocity control law is designed based on the Lyapunov function as follows:

(23) $$\left\{\begin{array}{l} v_{d}=v_{0}\cos(\theta -\alpha +\phi )\\ \omega_{d}=\omega_{r}-k\theta_{e} \end{array}\right.$$

The linear velocity error is defined as:

(24) $$e_{v}=v-v_{d}$$

where *v* is the current linear velocity of the tracked robot and *v_d_* is the desired linear velocity.

Construct the integral terminal sliding mode surface for the linear velocity error, *S_v_* [26]:

(25) $$S_{v}=e_{v}+\int_{0}^{t}\left(b_{1}{\left|\left.e_{v}\right|\right.}^{\gamma }\cdot {\mathrm{sgn}}^{\gamma }(e_{v})\right)dt$$

Take the derivative of the sliding mode surface:

(26) $${\dot{S}}_{v}={\dot{e}}_{v}+b_{1}|e_{v}{|}^{\gamma }\cdot {\mathrm{sgn}}^{\gamma }(e_{v})$$

Select the sliding mode reaching law:

(27) $${\dot{S}}_{v}=-b_{2}\cdot \mathrm{sgn}(S_{v})$$

For the dynamic model of the crawler robot in Equation (11):

(28) $$\left[\begin{array}{c} \dot{\nu }\\ \dot{\omega } \end{array}\right]=B\left[\begin{array}{c} u_{v}\\ u_{\omega } \end{array}\right]+\left[\begin{array}{c} f_{1}\\ f_{2} \end{array}\right]$$

where

$$B={\left[b_{1}\cdot u_{v},b_{2}\cdot u_{\omega }\right]}^{T},\left[\begin{array}{c} f_{1}\\ f_{2} \end{array}\right]=\left[\begin{array}{cc} a_{1} & 0\\ 0 & a_{2} \end{array}\right]\left[\begin{array}{c} \nu \\ \omega \end{array}\right]-\left[\begin{array}{c} -2\mu g\\ \frac{\mu mgL}{2J} \end{array}\right]+\left[\begin{array}{c} \zeta_{\nu }\\ \zeta_{\omega } \end{array}\right]$$

Let $x_{1}=y={\left[\begin{array}{c} v,\omega \end{array}\right]}^{T},x_{2}=D$ be the total disturbance of the system, and the system model can be expressed as:

(29) $$\left\{\begin{array}{l} {\dot{x}}_{1}=x_{2}+bu\\ {\dot{x}}_{2}=\delta \\ y=x_{1} \end{array}\right.$$

where *δ* is the derivative of the system disturbance *D*, and both *δ* and *D* are bounded, with the estimated value of *D* denoted as $\overset{\wedge }{D}$.

A nonlinear extended state observer (ESO) is designed to estimate the total disturbance of the system in real time.

(30) $$\left\{\begin{array}{l} e_{1}=z_{1}-y\\ {\dot{z}}_{1}=z_{2}-\beta_{1}e_{1}\\ {\dot{z}}_{1}=z_{3}-\beta_{2}fal(e_{1},a_{1},\delta_{1})+bu\\ {\dot{z}}_{3}=-\beta_{3}fal(e_{1},a_{2},\delta_{1}) \end{array}\right.$$

where *z*_1_ is the estimated value of *x*_1_, *z*_2_ is the estimated value of the total system disturbance, and *z*_3_ is the expanded variable of the observer, the estimated value of the total disturbance of the controller. *a*_1_ and *a*_2_ determine the nonlinearity of the fal function, where *a*_1_ is typically set to 5 and *a*_2_ to 0.25. *δ*_0_ represents the linear region width of the fal function around the origin; if the value is too large, it will reduce the ESO’s ability to estimate errors, while if it is too small, it may cause high-frequency oscillations. In this case, it is set to 0.1. *β*_1_ and *β*_2_ are the gains of the extended state observer, which influence the convergence speed of the ESO. By selecting appropriate gain values, the system’s disturbance estimation and compensation capabilities can be effectively enhanced.

The nonlinear function $\mathrm{fal}(x,a,\delta_{0})$ is defined as:

(31) $$\mathrm{fal}(x,a,\delta_{0})=\left\{\begin{array}{l} |x{|}^{a}\cdot \mathrm{sgn}(x),\;|x|>\delta \\ \frac{x}{{\delta_{0}}^{1-a}},\;|x|\leq \delta \end{array}\right.$$

As shown in Equation (31), the sgn function in the fal function is discontinuous at the piecewise points, which can easily cause chattering, amplify noise, and reduce the smoothness of control. Therefore, it is replaced by the Sigmoid function, which effectively smooths the transition, reduces chattering, and improves the system’s robustness and trajectory tracking accuracy. This approach is suitable for the operation of the tracked robot in non-flat environments.

(32) $$\mathrm{sign}(\varepsilon )=\frac{1}{1+e^{-\varepsilon }}-1$$

Substituting Equation (32) into Equation (31), the nonlinear function can be expressed as:

(33) $$sfal\left(\varepsilon ,a,\delta \right)=\left\{\begin{array}{l} |\varepsilon {|}^{a}(\frac{1}{1+e^{-\varepsilon }}-1)\;|\varepsilon |>\delta \\ \delta^{a}(\frac{1}{1+e^{-\varepsilon }}-1)\;|\varepsilon |⩽\delta \end{array}\right.$$

Based on the nonlinear error feedback control law (NLSEF), the linear velocity control law is designed as:

(34) $$u_{v}=k_{1}\cdot sfal(S_{v},a_{3},\delta_{1})+k_{2}\cdot sfal(e_{1},a_{4},\delta_{1})+k_{3}\cdot sfal({\dot{e}}_{1},a_{5},\delta_{1})-\frac{z_{3}}{b_{0}}$$

Similarly, the angular velocity control law is designed as:

(35) $$u_{\omega }={k_{1}}^{\prime }\cdot sfal(S_{\omega },a_{3},\delta_{1})+{k_{2}}^{\prime }\cdot sfal({e_{1}}^{\prime },a_{4},\delta_{1})+k_{3}\cdot sfal({{\dot{e}}_{1}}^{\prime },a_{5},\delta_{1})-\frac{{z_{3}}^{\prime }}{{b_{0}}^{\prime }}$$

In the control law, *a*_3_ generally taken as *a*_3_ = 0.75, *a*_4_ = 1.25, *a*_5_ = 1.5. The adjustment of *δ*_1_ is consistent with *δ*_0_ in the ESO. The parameter *k*_1_ mainly affects the tracking speed of the system, with larger values resulting in faster system response, while *k*_2_ mainly affects the anti-interference ability of the system, and increasing its value can suppress overshooting. *b*_0_ is determined by the system state equation. For a crawler robot operating in uneven environments where disturbances cannot be precisely modeled, *b*_0_ is treated as an adjustable parameter.

(36) $$V=\frac{1}{2}{S^{2}}_{v}$$

Take the derivative of it:

(37) $$\dot{V}=S_{v}{\dot{S}}_{v}$$

The dynamic error equation is:

(38) $${\dot{S}}_{v}=-k_{1}\cdot sfal(S_{v},a_{3},\delta_{1})-k_{2}\cdot sfal(e_{1},a_{4},\delta_{1})-k_{3}\cdot sfal({\dot{e}}_{1},a_{5},\delta_{1})+\frac{z_{3}}{b_{0}}$$

Substitute Equation (38) into Equation (37) to obtain:

(39) $$\dot{V}=S_{v}\left(-k_{1}\cdot sfal(S_{v},a_{3},\delta_{1})-k_{2}\cdot sfal(e_{1},a_{4},\delta_{1})-k_{3}\cdot sfal({\dot{e}}_{1},a_{5},\delta_{1})+\frac{z_{3}}{b_{0}}\right)$$

When $|\varepsilon |>\delta_{1}$, and $sfal\left(\varepsilon ,a,\delta_{1}\right)=|\varepsilon {|}^{a}(\frac{1}{1+e^{-\varepsilon }}-1)$ are substituted into Equation (37), we obtain:

(40) $$\begin{array}{l} \dot{V}=S_{v}\left(-k_{1}|S_{v}{|}^{a}\left(\frac{1}{1+e^{-S_{v}}}-1\right)-k_{2}|e_{1}{|}^{a}\left(\frac{1}{1+e^{-e_{1}}}-1\right)-k_{3}|\overset{\cdot }{e_{1}}{|}^{a}\left(\frac{1}{1+e^{-\overset{\cdot }{e_{1}}}}-1\right)+\frac{z_{3}}{b_{0}}\right)\\ \;\leq -k_{1}S_{v}|S_{v}{|}^{a}\left(\frac{1}{1+e^{-S_{v}}}-1\right)-k_{2}S_{v}|e_{1}{|}^{a}\left(\frac{1}{1+e^{-e_{1}}}-1\right)-k_{3}S_{v}|\overset{\cdot }{e_{1}}{|}^{a}\left(\frac{1}{1+e^{-\overset{\cdot }{e_{1}}}}-1\right)+\frac{z_{3}}{b_{0}} \end{array}$$

Since $\frac{1}{1+e^{-x}}-1$ is a monotonically increasing function and positive when *x* > 0, under the condition that *k*_1_, *k*_2_, *k*_3_ > 0, we have $\dot{V}\leq 0$.

When $|\varepsilon |⩽\delta_{1}$, substituting $sfal\left(\varepsilon ,a,\delta_{1}\right)={\delta_{1}}^{a}(\frac{1}{1+e^{-\varepsilon }}-1)$ into Equation (39) gives:

(41) $$\begin{array}{l} \dot{V}=S_{v}\left(-k_{1}{\delta_{1}}^{a}\left(\frac{1}{1+e^{-S_{v}}}-1\right)-k_{2}{\delta_{1}}^{a}\left(\frac{1}{1+e^{-e_{1}}}-1\right)-k_{3}{\delta_{1}}^{a}\left(\frac{1}{1+e^{-\overset{\cdot }{e_{1}}}}-1\right)+\frac{z_{3}}{b_{0}}\right)\\ \;\leq -k_{1}S_{v}{\delta_{1}}^{a}\left(\frac{1}{1+e^{-S_{v}}}-1\right)-k_{2}S_{v}{\delta_{1}}^{a}\left(\frac{1}{1+e^{-e_{1}}}-1\right)-k_{3}S_{v}{\delta_{1}}^{a}\left(\frac{1}{1+e^{-\overset{\cdot }{e_{1}}}}-1\right)+\frac{z_{3}}{b_{0}} \end{array}$$

Since $\frac{1}{1+e^{-x}}-1>0$, and *k*_1_, *k*_2_, *k*_3_ > 0, and $\dot{V}\leq 0$, according to Lyapunov stability theory, the system is asymptotically stable.

## 4. Trajectory Tracking Controller Parameter Adaptive Optimization

### 4.1. Improved Particle Swarm Optimization Algorithm

When a tracked robot performs trajectory tracking in an uneven terrain, external disturbances can cause changes in the curvature of the trajectory and abrupt variations in the heading angle, leading to deviations from the target trajectory. To address this issue, the robot must dynamically adjust its speed and heading angle based on real-time changes in the trajectory. In such a dynamic environment, the controller parameters must be adjusted in real time at different stages of operation to ensure that the robot remains stable along the desired path. To effectively solve this problem, a particle swarm optimization (PSO) algorithm is introduced to optimize the controller parameters.

The core idea of the traditional particle swarm optimization (PSO) algorithm is to find the global optimal solution by simulating the motion of particles (candidate solutions) in the search space and continuously optimizing their solutions. The velocity and position update of the i-th particle in the k-th iteration for each dimension d is determined by the following equations.

(42) $$\begin{array}{l} v_{i,d}(k+1)=w_{k}v_{i,d}(k)+c_{1}r_{1}(k)[p_{i,d}(k)-x_{i,d}(k)]+c_{2}r_{2}(k)[p_{g,d}(k)-x_{i,d}(k)]\\ x_{i,d}(k+1)=x_{i,d}(k)+v_{i,d}(k) \end{array}$$

where *i* is the particle index, *d* is the dimension index, and *k* is the iteration index. *v_i_*_,*d*_(*k*) and *x_i_*_,*d*_(*k*) represent the velocity and position of the *i*-th particle in the *d*-th dimension, respectively. *p_i_*_,*d*_(*k*) and *P_g_*_,*d*_(*k*) are the particle’s personal and global optimal positions in the *d*-th dimension, respectively. *w* is the inertia weight, controlling search dynamics, while *c*_1_ and *c*_2_ are learning factors guiding convergence. *r*_1_ and *r*_2_ are random numbers to ensure search diversity and avoid premature convergence. However, in the complex multi-modal optimization problem of trajectory tracking parameters for tracked robots in uneven terrain, the fixed inertia weight in the traditional PSO algorithm struggles to balance between global search and local optimization. To resolve this issue, this paper introduces particle similarity *s*(*i*,*j*) to monitor the changes in the state of the swarm.

(43) $$s(i,j)=\left\{\begin{array}{c} 1,\;d(i,j)<d_{\min}\\ 1-d(i,j)/d_{\max},\;d_{\min}⩽d(i,j)⩽d_{\max}\\ 0,\;d(i,j)>d_{\max} \end{array}\right.$$

A dynamic smoothing adjustment strategy is introduced based on particle similarity.

(44) $$\left\{\begin{array}{l} \omega (k)=\omega_{\max}-(\omega_{\max}-\omega_{\min})\cdot \frac{1+\tanh[s(i,j)-\varepsilon (k)]}{2}\\ \delta (k)=0.5\cdot \left(1-\frac{k}{N}\right) \end{array}\right.$$

where *W_max_* and *W_min_* are the maximum and minimum values of the inertia weight, respectively, *d*(*i*,*j*) is the Euclidean distance between particles *i* and *j*, *N* is the maximum number of iterations of the particle swarm, and *k* is the current iteration of the particle. This formula dynamically adjusts the inertia weight, balancing global and local search capabilities. It allows the particle swarm to quickly adapt to terrain changes while ensuring smooth transitions between stages, enhancing the algorithm’s adaptability for trajectory tracking in complex environments.

In non-flat terrain, the tracked robot’s controller must dynamically adjust parameters to adapt to complex conditions. The learning factors in the particle swarm algorithm determine the balance between individual and group learning, directly impacting optimization performance. Excessive individual learning leads to local optima and search divergence, while excessive group learning causes premature convergence to suboptimal solutions. To address this, a sine–cosine factor is introduced, enabling oscillatory decay within a fixed range. By probabilistically switching between sine and cosine functions, particle search diversity is enhanced, expanding the search space to optimize the controller for non-flat terrain. The adjustment formula is as follows:

(45) $$\left\{\begin{array}{l} c_{1}(k)=c_{1,\min}+(c_{1,\max}-c_{1,\min})\cdot \frac{1+\sin\left(\pi \cdot s(i,j)\right)}{2}\\ c_{2}(k)=c_{2,\min}+(c_{2,\max}-c_{2,\min})\cdot \frac{1+\cos\left(\pi \cdot (1-s(i,j))\right)}{2} \end{array}\right.$$

The fitness function of the algorithm is chosen as ITAE error criterion, the lower its value, the performance. The lower the value, the better the performance. The basic form is as follows:

(46) $$J=\int_{0}^{\infty }t\left|e_{1}(t)\right|+t|{e_{1}}^{\prime }(t)|dt$$

### 4.2. Tracking Controller Parameter Optimization Strategy

In this paper, an improved particle swarm optimization method is used to optimize the key parameters of the previously designed sliding mode self-immunity trajectory tracking controller, including *k*_1_, *k*_2_, *k*_3_,*b*_0_, *β*_1_, *β*_2_, and *β*_3._

The improved particle swarm optimization process is explained by the following pseudo-algorithmic code (see Algorithm 1).
**Algorithm 1 Improved Particle Swarm Algorithm Optimization Parameter Flow**  FOR each particle *i*   FOR each dimension *d*    Initialize position *x_i,d_*(*k*) randomly within permissible range    Initialize velocity v*_i,d_*(*k*) randomly within permissible range   END FOR  END FOR  Iteration *k* = 1  DO   FOR each particle *i*    Calculate the fitness value    IF the fitness value is better than *p_i,d_*(*k*) in history     Set current fitness value as the *p_i,d_*(*k*)    END IF   END FOR   Select the particle having the best fitness value as the *p_g,d_*(*k*)   FOR each particle *i*    FOR each dimension *d*     Update inertia weight *w*(*k*) according to the dynamic formula     Update learning factors *c*_1_(*k*), *c*_2_(*k*) using the sine–cosine adjustment formula     $\mathrm{Update}\;\mathrm{the}\;\mathrm{particle}\;\mathrm{velocity}\;\mathrm{using}\;\mathrm{the}\;\mathrm{equation}:\;v_{i,d}(k+1)=w_{k}v_{i,d}(k)+c_{1}r_{1}(k)[p_{i,d}(k)-x_{i,d}(k)]+c_{2}r_{2}(k)[p_{g,d}(k)-x_{i,d}(k)]$
     $\mathrm{Update}\;\mathrm{the}\;\mathrm{particle}\;\mathrm{position}\;\mathrm{using}\;\mathrm{the}\;\mathrm{equation}:\;x_{i,d}(k+1)=x_{i,d}(k)+v_{i,d}(k)$
    END FOR   END FOR  Update iteration count *k* = *k*+1  WHILE the maximum iterations or minimum error criterion are not attained

During the adjustment process, the variation range of the inertia weight is set as *w_max_* = 0.9 and *w_min_* = 0.4;. the variation range of the individual learning factor *c*_1_ is set as *c*_1,*min*_ = 0.5 and *c*_1,*max*_ = 2.5, with its value gradually decreasing with the number of iterations. Meanwhile, the variation range of the group learning factor *c*_2_ is set as *c*_2,*min*_ = 0.5 and *c*_2,*max*_ = 2.5. For the optimization of parameters *k*_1_, *k*_2_, *b*_0_, *β*_1_, *β*_2_, and *β*_3_, the particle dimension is set to 6; the particle swarm size is 100; the maximum number of particle iterations is 200; the upper limit of particle velocity is [1,1,0.25,1,2,5]; the lower limit of particle velocity is [−1,−1,−0.25,−1,−2,−5]; the upper limit of particle optimization is [400,400,30,200,1000,5000]; the lower limit of particle optimization is [1,1,1/30,60,200,1500]. 

**Figure 6:**
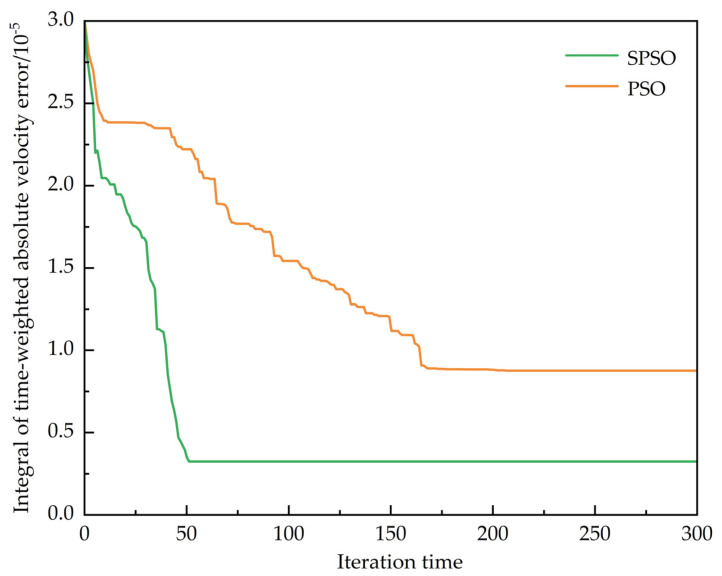
Comparison of fitness values between improved particle swarm optimization algorithm and classic particle swarm optimization algorithm.

Based on the previous theory, a simulation analysis of the improved particle swarm optimization (SPSO) algorithm was conducted; the result is shown in Figure 6. The performance of the improved SPSO and the classic particle swarm optimization (PSO) algorithm in terms of optimal fitness values showed significant differences. In the early stages of iteration, SPSO’s optimal fitness value dropped rapidly, indicating a faster convergence speed, while PSO’s decline was relatively slower. After 50 iterations, the SPSO curve leveled off, reaching the global optimum earlier. In contrast, PSO’s curve only started to stabilize after 153 iterations, and its final stable value was higher than that of SPSO, indicating that SPSO’s optimization results were superior to those of PSO. Additionally, the SPSO curve was smooth without oscillations, indicating stronger capability to escape local optima and more stable optimization, whereas the PSO curve exhibited fluctuations, reflecting its tendency to fall into local optima and insufficient optimization accuracy. SPSO, by dynamically adjusting the inertia weight and balancing global search with local exploration, not only significantly improved convergence speed and reduced the number of iterations but also consistently showed a smoother decline trend during iterations, maintaining stability after convergence. This fully demonstrated its efficiency and superiority.

## 5. Verification Experiment and Result Analysis

**Figure 7:**
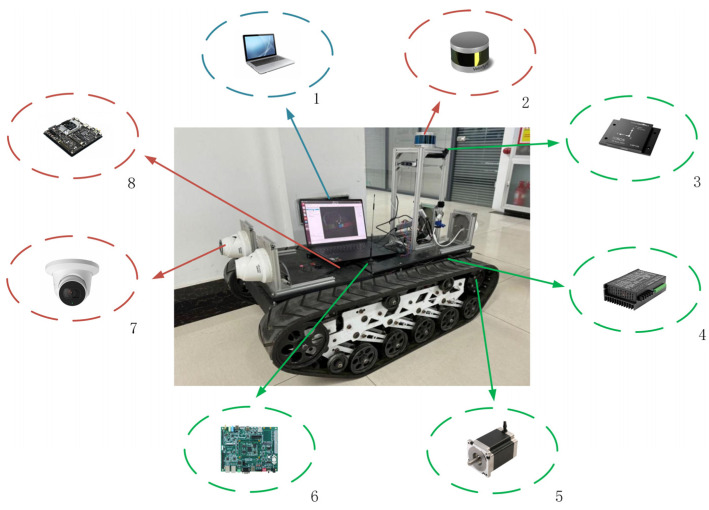
Crawler robot hardware structure. 1. Upper-level controller; 2. laser radar; 3. inertial sensor; 4. motor driver; 5. motor; 6. TrongLong M8 control board; 7. camera; 8. NVIDIA Jetson TX2 development board.

This paper uses a crawler robot as the experimental platform. The robot system mainly consists of the human–machine interaction layer, data processing layer, and control execution layer. The system hardware structure is shown in Figure 7.

The core of the human–computer interaction layer is the PC, which realizes remote communication with the robot through wireless network, and undertakes the functions of task issuance, parameter setting, status monitoring, etc. The data processing unit is based on the NVIDIA Jetson TX2 embedded computing platform and is equipped with powerful parallel computing capability of the GPU, receives task commands from the PC and acquires environmental information through LIDAR sensors and cameras to complete tasks such as map construction and robot localization. The sensing system consists of several types of sensors, including LIDAR for environment mapping and obstacle avoidance, a camera for visual recognition and target detection, an inertial measurement unit (IMU) for obtaining attitude and acceleration information, and an encoder mounted on the end of the motor shaft for measuring wheel speed and assisting in position estimation. The control part adopts the domestic TrongLong M8 control board, which integrates a CAN bus, serial port, and other communication interfaces, and is responsible for receiving motion commands and controlling the motor driver to generate control current to complete the differential motion, fixed-point steering, and other operations. The DC brushless motor is used as the power source for the drive mechanism, and the encoder is used to realize closed-loop control. The power supply of the system is provided by a 48 V lithium battery, and through the DC-DC step-down module for Jetson TX2, sensors, and other sub-systems, and at the same time is equipped with a power management unit, to achieve stable regulation of the voltage and current and fault protection, to ensure that the system operates stably over a long period of time. The technical parameters of the tracked robot are listed in Table 1, and the technical parameters of the DC brushless motor used in the robot’s drive wheels are listed in Table 2.

Based on the above technical parameters, the total rotational inertia of the tracked robot is *J* = 5.13 kg m^2^, and the rotational inertia of the wheels is *J_e_* = 1.05 kg m^2^. From this, the values *a*_1_ = −0.01, *a*_2_ = −0.23, *b*_1_ = 0.04, and *b*_2_ = 0.03.are derived. The steering resistance coefficient *μ* in the experimental environment is approximately 0.67.

**Figure 8:**
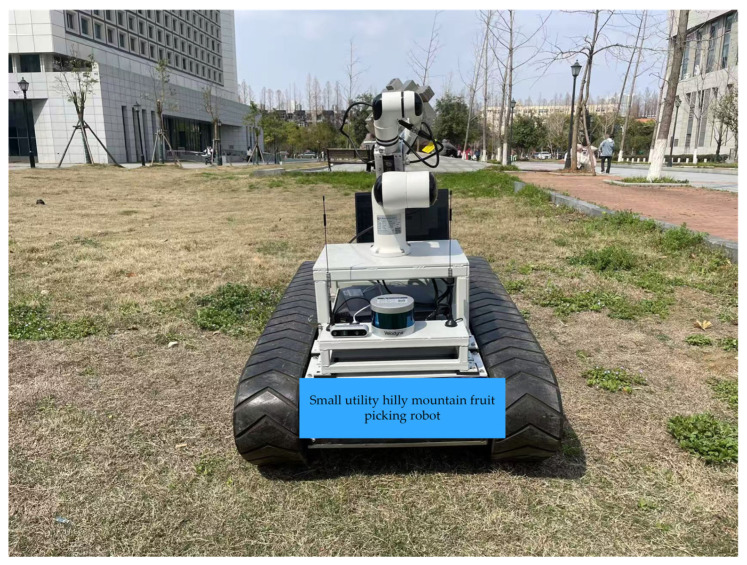
Experimental site.

To verify the trajectory tracking performance of the trajectory tracker designed in this paper, a path tracking experiment was conducted in an outdoor open area. The experimental area had dry grass covering the ground, with poor ground flatness and irregular depressions and protrusions, making the terrain complex. The experimental scene is shown in Figure 8. Considering the chassis performance and subsequent research requirements, the chassis forward speed was set to 0.5 m/s during the experiment. Comparative experiments were conducted on the tracked robot under three control methods: the improved particle swarm optimization-based sliding mode active disturbance rejection control (SPSMA), the classic particle swarm optimization-based sliding mode active disturbance rejection control (PSMA), and the traditional sliding mode active disturbance rejection control (SMA). The experiments involved tracking two arc-shaped trajectories with different curvatures.

### 5.1. U-Shaped Path Tracking Experimental Verification

This paper first selected the U-shaped path as the target trajectory. The path consists of straight lines and curves, simulating the common driving trajectory of a tracked robot during turning or obstacle avoidance in a non-flat environment. At the beginning of the experiment, the robot was stationary at the starting position, and the navigation control system was activated. After the system data stabilized, the robot started the trajectory tracking task and navigated along the U-shaped path. Considering the non-flat working environment when the tracked robot performs tasks, an initial lateral deviation of approximately 20 cm was set to verify the correction ability of the control algorithm. The traditional particle swarm algorithm and the improved particle swarm algorithm were used to optimize the parameters of the controller respectively, and compared with the unoptimized controller, the parameters of the sliding mode self-immunity trajectory tracking controller before and after optimization and when unoptimized are shown in Table 3.

**Figure 9:**
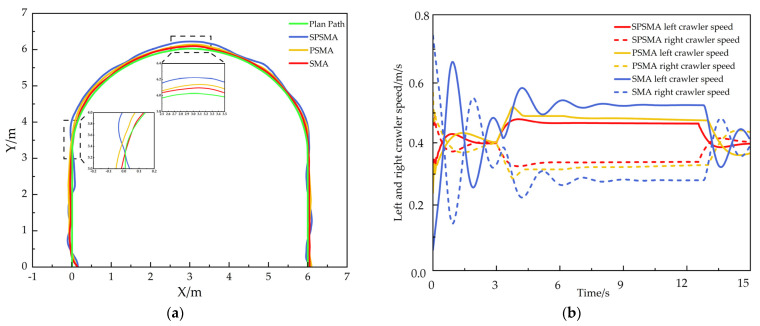
U-shaped trajectory tracking performance. (**a**) Overall trajectory tracking performance, (**b**) variation in left and right track speeds.

The U-shaped trajectory tracking results are shown in Figure 9. The experimental environment has an uneven ground with bumps and pits. All three control methods were capable of controlling the tracked robot to follow the U-shaped trajectory. However, the tracked robot controlled by the SMA method showed sensitivity to changes in the complex environment, with large deviations from the target trajectory on both sides of the track during the tracking process. The robot controlled by the PSMA method demonstrated good overall trajectory tracking performance, but still experienced some speed fluctuations and trajectory deviations during turns and on uneven surfaces. The robot controlled by the SPSMA method performed the best in the complex environment, with significantly reduced speed fluctuations on uneven surfaces and a trajectory that closely followed the target, minimizing the tracking deviation.

**Figure 10:**
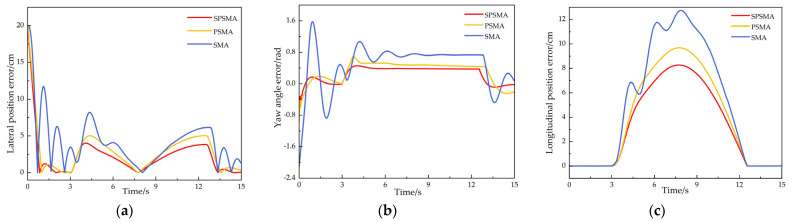
U-shaped trajectory tracking error. (**a**) Lateral position error, (**b**) yaw angle error, (**c**) longitudinal position error.

In the U-shaped trajectory tracking experiment, the actual position of the tracked track under different control methods was compared with the reference trajectory at each trajectory sampling point. The results are shown in Figure 10. The standard deviation of the position deviation was also calculated, as shown in Table 4. The position error index effectively reflects the trajectory tracking accuracy of the tracked robot in uneven environments, while the standard deviation reflects the driving stability of the tracked robot in such environments.

In order to better evaluate the overall tracking accuracy of the robot in complex trajectories, the navigation accuracy improvement percentage [21] was further used to measure the effectiveness of the navigation controller’s precision enhancement. The calculation formula is as follows:

(47) M=N1−N2/N1×100%

In the formula, *M* represents the navigation accuracy improvement percentage (%); *N*1 is the average deviation of the traditional controller (cm); and *N*2 is the average deviation of the improved controller (cm).

From Figure 10 and Table 3, the statistical analysis of the actual position deviation of the crawler robot at each trajectory sampling point compared to the reference trajectory shows that the SPSMA control method achieves a maximum lateral error of 4.02 cm, an average lateral error of 1.08 cm, a maximum longitudinal deviation of 7.81 cm, an average longitudinal error of 1.32 cm, a maximum position error of 8.28 cm, an average position error of 1.41 cm, a maximum yaw angle deviation of 0.56 rad, and a standard deviation of 3.19.

Compared to the PSMA control method, the SPSMA-controlled robot reduced the maximum lateral error by 24.58%, the average lateral error by 41.30%, the maximum longitudinal deviation by 14.18%, the average longitudinal error by 32.99%, the maximum position error by 19.22%, the average position error by 40.00%, the maximum yaw angle deviation by 28.21%, and the standard deviation by 20.65%.

Compared to the SMA control method, the performance improvement of the SPSMA-controlled robot is even more significant. The maximum lateral error decreased by 65.64%, the average lateral error decreased by 74.16%, the maximum longitudinal deviation decreased by 39.37%, the average longitudinal error decreased by 59.26%, the maximum position error decreased by 38.21%, the average position error decreased by 65.53%, the maximum yaw angle deviation decreased by 74.66%, and the standard deviation decreased by 42.63%

These results indicate that the SPSMA control method enables the robot to better respond to environmental changes, make real-time adjustments, reduce the accumulation of position errors, and show stronger adaptability to complex terrain and higher driving stability.

### 5.2. V-Shaped Path Tracking Experiment Validation

To further validate the improvement in track tracking accuracy of the crawler robot using the method proposed in this paper, positional information was pre-collected before the experiment, and a V-shaped trajectory tracking experiment with more significant curvature changes was conducted. The traditional particle swarm algorithm and the improved particle swarm algorithm were used to optimize the parameters of the controller respectively, and compared with the unoptimized controller, the parameters of the sliding mode self-immunity trajectory tracking controller before and after optimization and when unoptimized are shown in Table 5.

**Figure 11:**
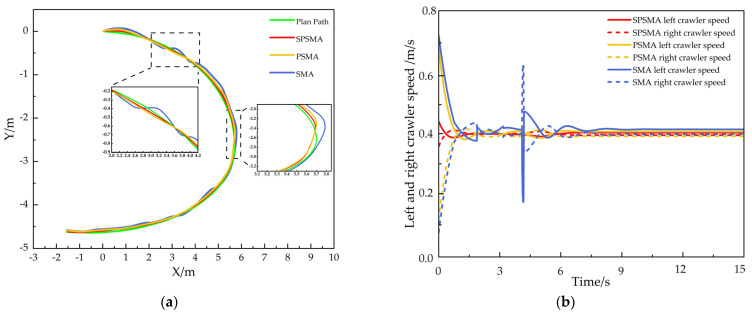
V-shaped trajectory tracking performance. (**a**) Overall trajectory tracking performance; (**b**) variation in left and right track speeds.

The experimental results are shown in Figure 11. The three control methods exhibited obvious differences in controlling the crawler robot’s trajectory tracking performance. The robot controlled by the SMA control method showed significant deviation from the trajectory during the early stage of the experiment, especially under terrain disturbances, and there were large fluctuations in speed when turning. The PSMA control method optimized the control parameters to partially suppress oscillations. Although the robot performed well in the initial stage of the trajectory, it still showed speed fluctuations and trajectory deviation in the turning areas, particularly where curvature changes were more pronounced. The SPSMA control method, on the other hand, effectively suppressed speed oscillations by further optimizing the control algorithm. It was able to quickly adjust the robot to the desired trajectory, even in the turning areas with significant curvature changes, maintaining minimal tracking deviation. The trajectory was smooth and stable, showing higher tracking accuracy and stronger environmental adaptability.

In the V-shaped trajectory tracking experiment, the actual position of the tracked track under different control methods was compared with the reference trajectory at each trajectory sampling point. The results are shown in Figure 10. The standard deviation of the position deviation was also calculated, as shown in Table 6. The position error index effectively reflects the trajectory tracking accuracy of the tracked robot in uneven environments, while the standard deviation reflects the driving stability of the tracked robot in such environments.

**Figure 12:**
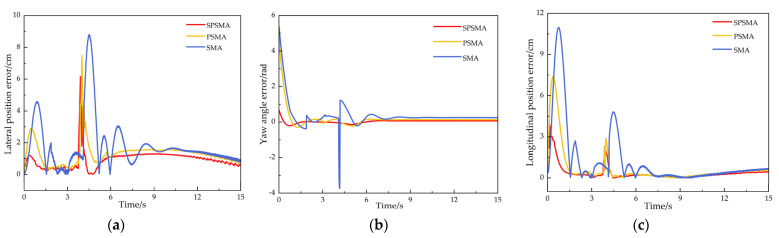
V-shaped trajectory tracking error. (**a**) Lateral position error, (**b**) yaw angle error, (**c**) longitudinal position error.

From the robot tracking error comparison in Figure 12 and the statistical tracking deviation in Table 6, it can be observed that the SPSMA control method controlled the tracked crawler robot’s maximum lateral error to 6.16 cm, with an average lateral error of 2.75 cm, a maximum longitudinal deviation of 3.95 cm, an average longitudinal error of 1.59 cm, a maximum position error of 9.26 cm, an average position error of 2.94 cm, a maximum yaw angle deviation of 0.87 rad, and a standard deviation of 4.28. Compared to the PSMA control method, the SPSMA control method reduced the robot’s maximum lateral error by 17.87%, average lateral error by 10.42%, maximum longitudinal deviation by 47.19%, average longitudinal error by 40.23%, maximum position error by 17.39%, average position error by 51.71%, maximum yaw angle deviation by 80.58%, and standard deviation by 25.68%.

In comparison with the SMA control method, the improvements of SPSMA were even more significant, with a reduction of the robot’s maximum lateral error by 30.86%, average lateral error by 37.78%, maximum longitudinal deviation by 66.16%, average longitudinal error by 49.04%, maximum yaw angle deviation by 37.71%, maximum position error by 39.85%, average position error by 52.04%, maximum yaw angle deviation by 84.49%, and standard deviation by 41.53%.

From the experimental results, it can be seen that the control method proposed in this paper shows better tracking accuracy than the traditional method in both the U-shaped trajectory and V-shaped trajectory tracking tasks of the crawler robot, and the overall control effect is remarkable. However, in the process of tracking the V-shaped trajectory, the maximum position error is still slightly higher than that of the U-shaped trajectory, which is related to the geometric characteristics of different paths, and there is an obvious sharp steering section in the V-shaped path, and the curvature change is abrupt, so the robot needs to complete the large-angle attitude adjustment in a short period of time. Since tracked robots rely on the rotational speed difference between the left and right tracks to realize steering, their structures are limited in their responsiveness when facing high curvature points, and are prone to steering lag. In addition, the transient nature of the attitude angle change also brings about a certain heading deviation, causing the robot to deviate from the preset trajectory for a short period of time, thus triggering a local error peak. In contrast, the curvature change of the U-shaped path is smoother, allowing the robot to have more sufficient response time to complete the attitude adjustment, and the error accumulation is relatively small.

Further analyzed from the perspective of dynamic characteristics, the tracked robot needs to overcome the significant inertia effect and asymmetric traction distribution when performing the sharp turn task. At the turning point of the V-shaped path, the inner and outer tracks are prone to slip and adhesion loss due to the difference in running paths and different force conditions, which, together with the sudden change in the angular momentum of the robot body, will lead to the intensification of dynamic perturbations in the attitude adjustment process. In addition, under the instantaneous steering condition, the nonlinear and coupled inertia terms within the system are significantly enhanced, which increases the difficulty of real-time compensation by the controller. In this study, the sliding mode self-resistant control strategy is adopted to improve the system’s disturbance-resistant performance and robustness, but there still exists a transient control lag at the point of sudden curvature change, which is manifested as an increase in the local error. In contrast, the continuity and curvature asymptotic properties of the U-shaped path help reduce the control load and enhance the smoothness of trajectory tracking.

## 6. Conclusions

The paper presents an improved particle swarm optimization (PSO) sliding mode self-perturbation (SPSM) control method for tracked robots to address trajectory deviation caused by uneven terrain. Using vector field guidance, the trajectory tracking problem is transformed into a heading control task, with the heading angle adaptively adjusted based on path curvature and position error. An extended state observer (ESO) was used to estimate disturbances in real-time, and a sliding mode self-perturbation controller was designed based on velocity error to dynamically correct the robot’s operational speed.

To address the issue of controller parameter optimization, the traditional PSO algorithm was improved by dynamically adjusting the inertia weight and updating learning factors, enhancing the algorithm’s search capability and convergence speed, optimizing control system parameters, and achieving precise trajectory tracking. Comparative experiments were conducted on U-shaped and V-shaped trajectories with different curvatures.

The results from the U-shaped trajectory tracking experiment show that the maximum position error of the SPSMA-controlled robot is 8.28 cm, the average position error is 1.41 cm, the maximum yaw angle deviation is 1.57 rad, and the standard deviation is 3.19. The proposed method in this paper achieves significant improvement in navigation tracking accuracy; the maximum position error of the robot is reduced by 19.22%, the average position error is reduced by 40.00%, and the heading angle error is reduced by 28.21%, as compared with the PSMA method, and compared with the SMA method, the maximum position error is reduced by 38.21%, the average position error is reduced by 65.53%, and the heading angle error is reduced by 74.66%.

In the V-trajectory tracking experiment, the maximum position error of the SPSMA-controlled robot is 9.26 cm, the average position error is 2.94 cm, and the maximum yaw angle deviation is 0.87 rad, with a standard deviation of 4.28. Compared with the PSMA-controlled method, the maximum position error, the average position error, and the heading angle error are reduced by 17.39%, 51.71%, and 80.58%, respectively. Compared with the SMA method, the maximum position error, mean position error and heading error are reduced by 38.95%, 52.04% and 84.49%, respectively.

Summarizing the results from both experiments, the SPSMA control method significantly improves the trajectory tracking accuracy of the tracked robot, effectively reduces trajectory tracking error fluctuations, and enhances tracking stability. The method demonstrates superior trajectory tracking performance in complex, uneven environments, providing technical support for high-precision navigation.

## Figures and Tables

**Table 1:** Tracked robot technical parameters.

Parameters	Values
Maximum motor power/Kw	1.5 × 2.0
Mass/kg	105
Overall dimensions/m × m × m	1.1 × 0.75 × 0.3
Track gauge/m	0.5
Pitch/m	0.7
Drive wheel diameter/m	0.18
Track grounding length/m	0.7
Maximum forward speed/(m·s^−1^)	1.2
Drive efficiency	0.89
Gear ratio	30

**Table 2:** Motor technical parameters.

Parameters	Values	Unit
Rated power	1.5 × 2.0	Kw
Rated torque	4.8	N·m
Rated speed	3000	rpm
Rated voltage	48	V
Rated current	39	A
Back emf	8.8	V
Motor coil resistance	0.2	Ω
Torque constant	0.12	N·m/A
Voltage constant	0.003	V·s/rpm
Rotor inertia	11 × 10⁻⁴	kg·cm^2^

**Table 3:** Controller optimization parameters before and after comparison.

Controllers	Parameters	Pre-Optimization	Classical Particle Swarm Optimization	Improved Particle Swarm Optimization
Linear speed control	*k*_1_	120	178.2	263.7
	*k*_2_	80	118.6	175.5
	*k*_3_	50	74.2	109.8
	*b*_0_	20	24.1	28.6
	*β*_1_	100	145.0	218.3
	*β*_2_	450	662.5	880.12
	*β*_3_	3000	4420.0	4800.0
	*k*_1_	120	177.5	262.9
Angular velocity control	*k*_2_	100	147.6	218.8
	*k_3_*	50	83.1	123.1
	*b*_0_	20	24.0	28.5
	*β*_1_	100	144.2	216.9
	*β*_2_	450	559.0	785.3
	*β*_3_	3000	4310.7	4935.2

**Table 4:** Robot U-shape trajectory tracking positioning error.

Controller	Max Lateral Position Error (cm)	Avg Lateral Position Error (cm)	Max Longitudinal Position Error (cm)	Avg Longitudinal Position Error (cm)	Max Position Error (rad)	Avg Position Error (rad)	Max Yaw Angle Error (rad)	Standard Deviation
SPSMA	4.02	1.08	7.81	1.32	8.28	1.41	0.56	3.19
PSMA	5.33	1.84	9.10	1.97	10.25	2.35	0.78	4.02
SMA	11.7	4.18	12.88	3.24	13.40	4.09	2.21	5.56

**Table 5:** Controller optimization parameters before and after comparison.

Controllers	Parameters	Pre-Optimization	Classical Particle Swarm Optimization	Improved Particle Swarm Optimization
Linear speed control	*k*_1_	120	331.72	343.45
	*k*_2_	100	323.52	353.22
	*k_3_*	60	215.45	236.07
	*b*_0_	20	24.08	28.63
	*β*_1_	100	115.13	130.27
	*β*_2_	450	660.19	782.38
	*β*_3_	3000	3600	4000
Angular velocity control	*k*_1_	120	231.44	342.43
	*k*_2_	100	225.18	335.36
	*k_3_*	60	125.41	186.22
	*b*_0_	20	23.51	26.53
	*β*_1_	100	113.69	128.15
	*β*_2_	450	670.38	783.29
	*β*_3_	3000	3600	4200

**Table 6:** Robot V-shape trajectory tracking positioning error.

Controller	Max Lateral Position Error (cm)	Avg Lateral Position Error (cm)	Max Longitudinal Position Error (cm)	Avg Longitudinal Position Error (cm)	Max Position Error (rad)	Avg Position Error (rad)	Max Yaw Angle Error (rad)	Standard Deviation
SPSMA	6.16	2.75	3.95	1.59	9.26	2.94	0.87	4.28
PSMA	7.50	3.07	7.48	2.66	11.21	1.47	4.48	5.76
SMA	8.91	4.42	11.67	3.12	14.72	6.13	5.61	7.32

## Data Availability

Data are contained within the article.
